# Daily Treatment with SMTC1100, a Novel Small Molecule Utrophin Upregulator, Dramatically Reduces the Dystrophic Symptoms in the *mdx* Mouse

**DOI:** 10.1371/journal.pone.0019189

**Published:** 2011-05-06

**Authors:** Jonathon M. Tinsley, Rebecca J. Fairclough, Richard Storer, Fraser J. Wilkes, Allyson C. Potter, Sarah E. Squire, Dave S. Powell, Anna Cozzoli, Roberta F. Capogrosso, Adam Lambert, Francis X. Wilson, Stephen P. Wren, Annamaria De Luca, Kay E. Davies

**Affiliations:** 1 Summit plc, Abingdon, United Kingdom; 2 MRC Functional Genomics Unit, Department of Physiology Anatomy and Genetics, University of Oxford, Oxford, United Kingdom; 3 Unit of Pharmacology, Department of Pharmaco-biology, University of Bari “A. Moro”, Bari, Italy; Virginia Commonwealth University, United States of America

## Abstract

**Background:**

Duchenne muscular dystrophy (DMD) is a lethal, progressive muscle wasting disease caused by a loss of sarcolemmal bound dystrophin, which results in the death of the muscle fibers leading to the gradual depletion of skeletal muscle. There is significant evidence demonstrating that increasing levels of the dystrophin-related protein, utrophin, in mouse models results in sarcolemmal bound utrophin and prevents the muscular dystrophy pathology. The aim of this work was to develop a small molecule which increases the levels of utrophin in muscle and thus has therapeutic potential.

**Methodology and Principal Findings:**

We describe the *in vivo* activity of SMT C1100; the first orally bioavailable small molecule utrophin upregulator. Once-a-day daily-dosing with SMT C1100 reduces a number of the pathological effects of dystrophin deficiency. Treatment results in reduced pathology, better muscle physiology leading to an increase in overall strength, and an ability to resist fatigue after forced exercise; a surrogate for the six minute walk test currently recommended as the pivotal outcome measure in human trials for DMD.

**Conclusions and Significance:**

This study demonstrates proof-of-principle for the use of *in vitro* screening methods in allowing identification of pharmacological agents for utrophin transcriptional upregulation. The best compound identified, SMT C1100, demonstrated significant disease modifying effects in DMD models. Our data warrant the full evaluation of this compound in clinical trials in DMD patients.

## Introduction

Duchenne muscular dystrophy (DMD) is a lethal X-linked recessive muscle wasting disease caused by mutations in the dystrophin gene (for review see [1]). Affected boys are ambulatory until about 12 years of age but often live into their twenties with recent improvements in respiratory support. Many boys show an abnormal ECG in the late stages of the diseases and cardiomyopathy is also a general feature. The milder form of the disease known as Becker muscular dystrophy (BMD) is also characterized by cardiac defects despite BMD patients often being ambulant in their 50s and 60s. Thus, any therapy for the disease would need not only to target skeletal, but also cardiac muscle.

Currently there is no effective treatment for DMD. Various strategies developed to alleviate the symptoms include steroid treatment, anti-inflammatory agents, and growth hormone and myostatin inhibitors (for review see [2]). More recently, genetic approaches have been tested in DMD patient trials. In particular, readthrough of stop codons has been attempted in the 10–15% of patients that have mutations resulting in premature stop codons resulting in dystrophin deficiency. An orally delivered small molecule, Ataluren, recently entered a phase IIb trial. The six minute walk distance test [3] (6MWD) was used as the primary efficacy endpoint as the ability to walk further after treatment is considered by the regulatory authorities as a major improvement in the quality of life for these patients. Unfortunately, after conclusion of the trial, no statistically significant increase in the distance travelled using the 6MWD was reported. Skipping of exon 51, which targets up to 13% of patients, represents the monoskipping therapy which would be applicable to the largest proportion of DMD patients. Antisense molecules, delivered either intravenously or sub-cutaneously, have shown some restoration of dystrophin to a variable degree in patients [4], [5]. Next generation trials are planned with constructs which increase the efficiency of delivery of the antisense oligonucleotides. The efficacy of this approach was demonstrated using the dystrophin/utrophin knock-out mouse, where restoration of muscle function was demonstrated [6]. To treat more patients, different antisense sequences will need to be developed to target other exons and the regulatory authorities may treat each of these new constructs as a new drug. The ideal scenario would be to develop multi-exon skipping [7] but this may only be achieved using AAV delivery which faces immunological problems.

We have taken an alternative pharmacological approach to DMD by developing an orally bioavailable small molecule which should be appropriate to treat all patients irrespective of their mutation and target both skeletal and cardiac muscle. Building on our work in the *mdx* mouse, which demonstrated that the loss of dystrophin could be compensated for by increasing the levels of the dystrophin-related protein, utrophin, we have developed novel small molecules which can transcriptionally upregulate the utrophin gene. The demonstration that increased utrophin can reduce the muscular dystrophy in the *mdx* mouse has been confirmed by others [8]–[11]. Our early data from the *mdx* mouse suggested that increasing the levels of utrophin over two-fold would be of great benefit [12].

SMT C1100 was the final product of an exhaustive chemical screening and optimisation campaign. In this paper we present evidence confirming an overall two-fold increase in both utrophin RNA and protein resulting in a significant reduction in dystrophic symptoms and increased muscle function in dystrophin-deficient *mdx* mice. This was a comprehensive study looking at the beneficial effects of daily dosing of SMT C1100 in both sedentary *mdx* and the more severely affected forced exercise model. If the results obtained here using SMT C1100 translated across to DMD patients then undoubtedly this would be a disease modifying therapy for DMD.

## Methods

### Ethics Statement

All animal procedures were performed in accordance with UK Home Office regulations or in accordance with the Italian Guidelines for the use of laboratory animals, which conform with the European Community Directive published in 1986 (86/609/EEC). The work performed in Oxford was performed under certificate of designation number 30/2306 and project license number 30/2652 following approval by the University of Oxford Departments of Physiology, Anatomy & Genetics and Experimental Psychology Joint Departmental Ethics Review Committee. The work performed by Covance Laboratories Ltd. was performed under certificate of designation number 50/8504 and project license number 60/3774 following approval by the Covance Ethical Review Process. The work in Bari was approved by the central review board under the Italian Minister of Health which authorizes that all animal studies conform to the ethical requirement and current law.

### Generation of a utrophin promoter screening cell line

Murine H2K cells [13] were transfected with 8.4 kb of the human utrophin promoter including the first untranslated exon linked to a luciferase reporter gene and stable lines generated for screening (H2K-*mdx* utrnA-luc).

### Cell culture

The H2K-*mdx* utrnA-luc cells were maintained in DMEM (Invitrogen) supplemented with 20% Fetal Bovine Serum Gold (PAA), 5% CEE (SLI), 2 mM L-Glutamine (Invitrogen), 1% Penicillin Streptomycin (Invitrogen) and 2 µg/500 ml Mouse Interferon-γ (Roche). Cells were maintained at 10% CO_2_ at 33°C. Normal human skeletal muscle cells (SkMc) were sourced from TCS cell works. Passaging was undertaken according to the supplier's instructions including the use of specialist culture media. Cells were maintained at 5% CO_2_ at 37°C.

### *In vitro* assays

For luciferase assays plates were seeded with H2K-*mdx* utrnA-luc at 5000 cells per well. These were incubated in 10% CO_2_ and 33°C for 24 h prior to dosing.

All compounds were supplied as a 10 mM solution in dimethyl sulfoxide (DMSO). Cells were treated compounds dissolved in a final concentration of 0.3% DMSO. Assays were performed in triplicate and the compounds were dosed for 48 h. Luciferase levels were measured using the Steady-Glo Luciferase kit (Promega) and the plates were then read using a FLUOstar Optima plate reader (BMG Labtech). The means from the biological triplicates were used in a 4 Parameter Logistic Model to generate an EC_50_ and Hill slope. The software used to calculate this was XLfit version 4.2.2.

### Sedentary mice and drug treatment

Three week-old male *mdx* (C57BL/10ScSn-Dmd*^mdx^*/J; Charles River Laboratories, MA, USA) littermates were randomly split between 2 groups and treated with SMT C1100 (50 mg/kg) or vehicle only (phosphate buffered saline (PBS), 0.1% Tween-20, 5% DMSO) *via* daily i.p. injection for four weeks. At the end of the drug treatment period mice were sacrificed by CO_2_ asphyxiation in accordance with Schedule I of the UK Animals (Scientific Procedures) Act 1986. C57BL/6 contractile properties were measured in EDL muscle dissected from eight week old untreated mice obtained at 4 week of age from Harlan (n = 5). All animal procedures were performed in accordance with UK Home Office regulations. In all other experiments described using the sedentary *mdx* mice dosing was by the oral gavage using a canula to deliver SMTC1100 or vehicle only on a daily basis for 28 days. At the end of this period the mice were sacrificed and muscle and blood samples were taken. Quantification of muscle and plasma levels of SMT C1100 was performed using CD1 mice.

### Exercised mice, treadmill running and drug treatment

Most of the experimental procedures conform to the standard operating procedures for pre-clinical tests using *mdx* mice available at http://www.treat-nmd.eu/research/preclinical/SOPs/.

A total of 24 *mdx* male mice of 4–5 weeks of age (Charles River-Italy), and homogeneous for body weight, underwent a 30 min running regime on an horizontal treadmill (Columbus Instruments) at 12 m/min, twice a week (keeping a constant interval of 2–3 days between each trial), for 4–6 weeks, according to a standard protocol [13], [14]. Experimental groups were treated as follows: vehicle only (n = 7), SMT C1100 (50 mg/kg; n = 6), α-methyl prednisolone (PDN; 1 mg/kg; n = 5) and combination of SMT C1100 (50 mg/kg) and PDN (1 mg/kg) (n = 6). Age and gender-matched wild type C57/BL10ScSn) or non-exercised *mdx* mice were also used for specific experimental purposes, as indicated in the text. The dose of PDN has been chosen based on our previous studies [14]. The treatment started one day before the beginning of the exercise protocol, and continued until the day of sacrifice. SMT C1100 and the combination of PDN+SMT C1100 were dissolved in 5% DMSO, 0.1% Tween-20 in PBS, whilst PDN was dissolved in sterile water. Drugs were formulated for i.p. injection so that the correct dose was administered in 0.1 ml/10 g. Body weight was assessed weekly, as was fore-limb force by means of a grip strength meter (Columbus Instruments, USA) [14], [15]. An exercise resistance test, consisting of horizontal running for 5 min at 5 m/min, then increasing the speed of 1 m/min each minute, was performed on week 0 and after four and five weeks of treatment. The total distance run by each mouse until exhaustion was measured [15]. At the end of the 5^th^ week of exercise/treatment the *ex vivo* experiments were also started. To this aim mice were deeply anesthetized and sacrificed using 1.2 g/kg urethane (i.p.) in accordance with the Italian Guidelines for the use of laboratory animals, which conform with the European Community Directive published in 1986 (86/609/EEC).

### Muscle mechanics/electrophysiology

Muscle mechanics were conducted on the extensor digitorum longus (EDL) muscle as previously described [6]. EDL muscles were removed and rapidly placed in the recording chamber for the electrophysiological or isometric recordings. In the recording chambers EDL muscles were bathed at 30±1°C in the following normal physiological solution: NaCl 148 mM, KCl 4.5 mM, CaCl_2_ 2.0 mM, MgCl_2_ 1.0 mM, NaHCO_3_ 12.0 mM, NaH_2_PO_4_ 0.44 mM, glucose 5.55 mM, and continuously gassed with 95% O_2_ and 5% CO_2_ (pH 7.2–7.4). The mechanical threshold (MT) was determined in EDL muscle fibres by the two microelectrode “point” voltage clamp method, described previously [13], [14]. In brief, depolarizing command pulses of variable duration (from 500 to 5 ms at 0.3 Hz) were progressively increased in amplitude from the holding potential (H) of −90 mV until a visible fiber contraction. The mean threshold membrane potential values of individual myofibers (V, in mV) at various pulse durations (t, in ms) allowed the construction of a “strength-duration” curve. The rheobase voltage (R, in mV) was obtained by a non-linear least square algorithm using a previously described equation [14], [15].

### Protein analyses

A human DMD cell line with a deletion of exons 49 and 50 (generously provided by Vincent Mouly, Paris) was seeded in 6 cm dishes in DMEM (Invitrogen), Medium 199 (20%; Sigma-Aldrich), Fetal Calf Serum (20%; Invitrogen), glutamine and penicillin/streptomycin (Invitrogen) 24 h prior to drug treatment. After three days of drug treatment cells were lysed for 20 min at 4°C in Tris pH6.8 (75 mM), SDS (3.8%), Urea (4 M), glycerol (20%) supplemented with protease inhibitors (1∶100; Sigma-Aldrich). Soluble protein was purified at 8000×*g* for 20 min at 4°C and 30 µl was size fractionated by 5% Tris-HCL SDS-PAGE gel electrophoresis and transferred to a PVDF membrane (GE Healthcare). Soluble protein was prepared from muscle samples snap frozen in liquid nitrogen and stored at −80°C until use for biochemical analysis. For western blotting, protein was crushed with a pestle in a liquid nitrogen-cooled mortar, solubilised in 50 volumes of single-section western blot lysis buffer [16], vortexed, briefly homogenised and sonicated, heated to 94°C for 4 minutes and centrifuged for 3 min at 15 000×*g* to remove insoluble matter. For western blotting, 50 µl soluble protein extract was separated by 5% Tris-HCL SDS-PAGE gel electrophoresis and transferred onto PVDF (GE Healthcare). Utrophin protein was detected using MANCHO 3 antibody (1∶100; kind gift from G.E. Morris, Oswestry, UK) and ECL HRP-conjugated anti-mouse antibody (GE Healthcare). Equal protein loading was corrected by detection of α-actinin (1∶200, N-19; Santa Cruz Biotechnology, Inc.) and a HRP-conjugated anti-goat antibody (Sigma). Blots were developed using ECL reagent (GE Healthcare), Densitometry was performed using the freely available web version of Image J (rsbweb.nih.gov/ij/). Immunohistochemistry was carried out as previously described [17].

### RNA analyses

For quantitative real time RT-PCR SkMC cells were seeded in six well plates at 25000 cells per well in 3 ml of appropriate media and incubated for 24 h prior to dosing. Compounds were dosed in a final concentration of 1% DMSO for 72 h. RNA was extracted from tissue using a QIAGEN RNeasy® Plus kit (Qiagen) and QiaShredder (Qiagen), using the manufacturer's instructions. The High-Capacity cDNA Reverse Transcriptase Kit (Applied Biosystems) was used according to manufacturer's instructions. Real-time PCR was performed according to the ΔΔCT method [18]. The 7300 Real-Time PCR System from Applied Biosystems was used for this assay along with the 7300 System SDS software with the SDS Relative Quantification Study Plug in. Data was analysed using the 7300 System SDS software with the SDS Relative Quantification Study Plug in.

### Blood analyses

Measurements of SMT C1100 plasma concentrations were obtained following RO (retro-orbital) blood sampling on day 1, 24 h after oral dosing with SMT C1100 (50 mg/kg) or vehicle only (0.1% PBS-Tween-20/5% DMSO). Further samples were taken at day 15 and day 28 after the start of dosing.

Blood was collected with non-heparinized hematocrit tubes into serum microtainer tubes and centrifuged for 12 min at 12,000 rpm at 4°C. Serum was stored at −80°C prior to analysis using the CK (NAC) reagent kit in conjunction with the AU 400 Clinical Chemistry analyser (Olympus UK Ltd).

### Histological analyses

After 28 days of dosing muscle samples were taken for histopathology and processed by Premier Laboratory LLC (Colorado, U.S.A.). Samples were received in 10% neutral buffered formalin, processed into paraffin, and 5 µm sections and stained for Hematoxylin and Eosin (H&E) and Masson's Trichrome (tibialis anterior, extensor digitorum longus, soleus, and diaphragm). Both the H&E and Masson's Trichrome stained slides were submitted blind to a Board Certified Veterinary Pathologist at Premier Laboratory LLC and scored for the presence of inflammation and fibrosis. A total of five sections from each muscle were analysed.

The muscle fibres were scored according to the following criteria:

Inflammation:
  0 = none to minimal - No inflammation within the muscle bundles or inter-bundle connective tissue; occasional mononuclear inflammatory cells may be present but no obvious aggregations.
  1 = mild - Occasional mononuclear inflammatory cells in the inter-bundle connective tissue with focal aggregations of mononuclear inflammatory cells.
  2 = moderate - Multiple foci of mononuclear inflammatory cell infiltration in the inter-bundle connective tissue; occasional mononuclear inflammatory cells between individual muscle fibres.
  3 = severe - Multiple large foci of mononuclear inflammatory cell infiltration in the inter-bundle connective tissue extending into the intra-bundle connective tissue with expansion of the inter-bundle and intra-bundle space.
Fibrosis:
  0 = none to minimal - No fibrosis in the muscle bundles or inter-bundle connective tissue; mild expansion of the inter-bundle connective tissue may be present focally.
  1 = mild - Focal expansion of the inter-bundle connective tissue; mild focal expansion of the intra-bundle space may be present.
  2 = moderate - Multiple foci of expansion of the connective tissue component in the inter-bundle area; focal intra-bundle increases in connective tissue between individual muscle fibres.
  3 = severe - Multiple large foci of connective tissue in the inter-bundle region extending into the intra-bundle connective tissue with expansion of the inter-bundle and intra-bundle space.

In addition two 42-bit color images were captured with a Zeiss AxioCam HR digital camera on a Zeiss Axioskop 2 microscope utilizing AxioVision 4.4 software (Zeiss) of each muscle on the H&E stained slides. Once the images were captured they were white balanced in Adobe Photoshop (Adobe). The proportion of centrally nucleated fibers was determined by analyzing the images and counting the number of centrally located nuclei; a total of two hundred cells per muscle were evaluated. Students' two-tailed t-test was used to compare the groups with significance set at p<0.05.

### Statistical Analyses

Significance was calculated using the Student's t test with a two-tailed distribution assuming unequal sample variance. Multiple statistical comparisons between groups, was performed by one-way ANOVA, with Bonferroni's t test post-hoc correction for allowing a better evaluation of intra- and inter-group variability and avoiding false positive.

## Results

### *In vitro* upregulation of utrophin

**Figure 1:**
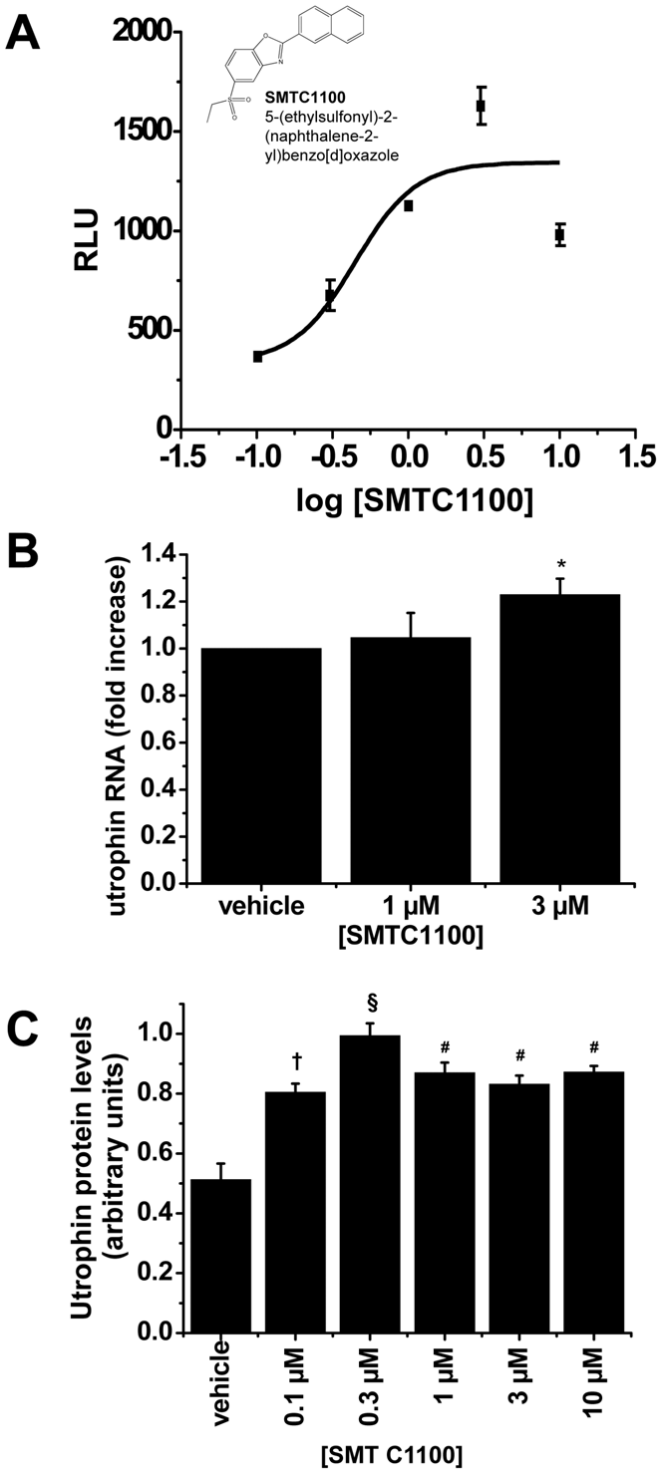
*In vitro* activity of SMT C1100. (A) SMT C1100 dose response in murine *H2k-mdx* utrnA-luc cells expressing the human utrophin promoter linked to a luciferase reporter gene. Cells were treated with compound for 48 h in standard growth medium containing 0.3% DMSO. The chart shows relative luminescence (RLU) in relation to five different doses of SMT C1100. A Four Parameter Logistic Model was used to generate an EC_50_. Points represent a mean ±S.E. of three experiments and are typical of the results for all batches of SMTC1100. The structure for SMTC1100 is shown; (B) SMT C1100 significantly increased mRNA copy number of the utrophin transcript in SkMC cells. In this assay Gene Expression Assay 4326315 was used for β-actin detection and Gene Expression Assay Hu01125984_m1 was used for utrophin transcript detection (both Applied Biosystems). Cells were exposed to SMT C1100 in standard media with 1% DMSO (vehicle) for 72 hours with six biological replicates. *p = 0.01 relative to vehicle only; (C) Utrophin protein levels in human DMD cell line treated with SMT C1100 (1 µM) or vehicle (0.1% DMSO). Blots were stained with anti-utrophin (MANCHO3; 1∶100) and ECL HRP-conjugated anti-mouse antibody (GE Healthcare). Bands were quantified using Image J and arbitrary units represent utrophin levels corrected for equal loading by α-actinin immunostaining. Results represent a mean ± S.E based on n = 3. †p = 0.00683; §p<0.001; #p<0.005 relative to vehicle-treated cells.

SMT C1100 was identified from an iterative analoging approach from initial hits identified using a human muscle specific utrophin A promoter cell-based assay. Myoblasts (*mdx*) were cloned from H-2K-tsA58×*mdx* with an IFN/tsSV40 T-Ag transgene in order to control proliferation and fusion [13]. The screening line named H2K-*mdx* utrnA-luc contains a stably integrated reporter consisting of 8.4 kb of the human utrophin promoter linked to a luciferase reporter gene. The region of the utrophin promoter contained all the motifs known to control utrophin expression [19], [20]. This high throughput screening assay identified a number of luciferase-inducing compounds that also have the ability to increase the transcription of the endogenous mouse *UTRN*, thus identifying compounds with both human and mouse activity eventually leading to the final optimized compound, SMT C1100 whose chemical structure is shown in Fig. 1A.

SMT C1100 shows a maximal increase of four to five-fold compared to vehicle with an EC_50_ of 0.4 µM (Fig. 1A). *In vitro* dosing of human myoblasts with SMT C1100 leads to a 25% increase in utrophin mRNA (Fig. 1B) when compared to vehicle-only dosing after three days of treatment. Treatment of human DMD cells with SMT C1100 lead to a 2-fold increase in utrophin protein levels at an optimal concentration of 0.3 µM after 3 days of treatment (Fig. 1C).

### Plasma levels of SMT C1100

**Figure 2:**
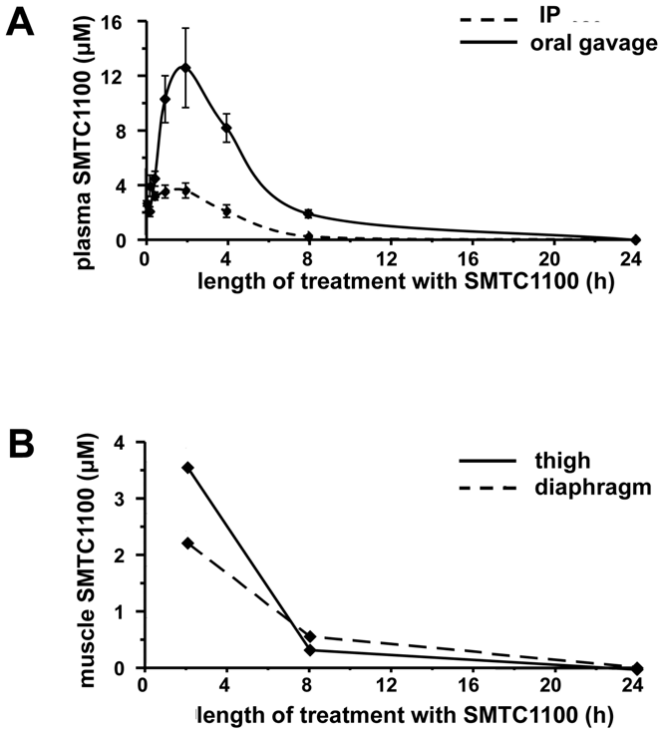
Plasma levels of SMT C1100 in the mouse. (A) SMT C1100 plasma levels were assessed over a 24 h period after oral gavage or i.p. delivery of 50 mg/kg of the compound into wild type CD1 mice. At set time points after administration groups of three animals were taken for blood samples at the times stated in the figure. (B) Thigh and diaphragm samples from CD1 dosed orally with 50 mg/kg were quantified for the presence of SMT C1100.

Significant plasma (Fig. 2A) and muscle (Fig. 2B) levels of compound were achieved for several hours following oral administration of SMT C1100 (50 mg/kg). From the EC_50_ data (Fig. 1A), taken together with the levels of utrophin upregulation achieved at various drug concentrations in both DMD and normal myoblasts, we can estimate that the effective concentration required for efficacy would be in the order of 0.5–1 µM. This means that therapeutic levels are achieved in muscle for at least eight hours following dosing.

### Toxicological evaluation of SMT C1100

In order to confirm that SMT C1100 had no obvious off target toxicological liabilities mice were dosed with high levels of compound. Overall no toxicologically significant changes in clinical condition, body weight, food consumption, haematology or clinical chemistry parameters were seen during the study. There were no microscopic findings within the comprehensive set of tissues analysed due to effects of SMT C1100. Conclusion from Covance Laboratories Ltd. confirmed that the study did not identify any toxicity that was attributable to dosing with SMTC1100. Based on the conditions of this study, it was considered that no toxicity was determined for SMT C1100 administered by oral gavage to the mouse up to dose levels of 2000 mg/kg/day for 28 days. This information was a key component of the toxicology assessment which led to a successful clinical trial application and testing in healthy volunteers. This was only one component of a significant package of safety evaluation performed on SMT C1100.

### *In vivo* upregulation of utrophin

**Figure 3:**
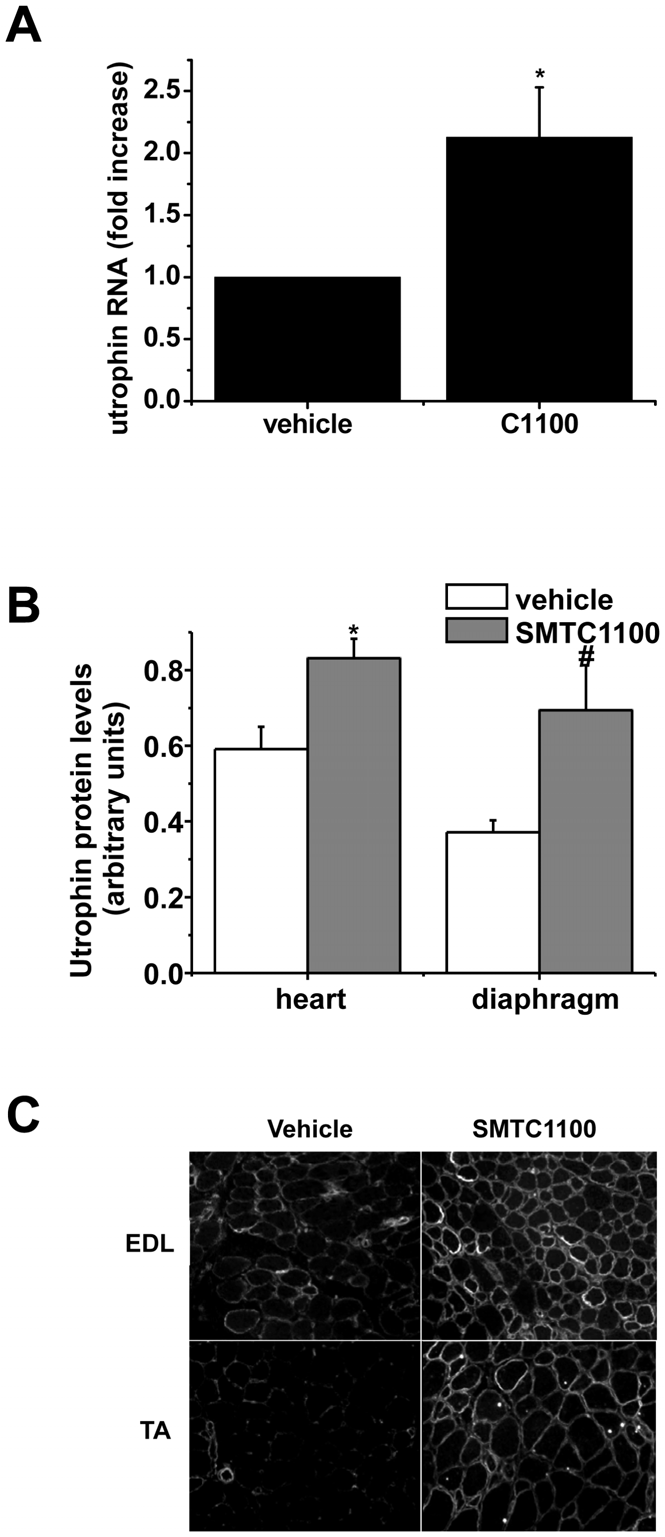
Effect of SMT C1100 on *in vivo* utrophin levels in the *mdx* mouse. (A) Two-fold increase in utrophin mRNA following daily oral administration of *mdx* mice with SMT C1100 (50 mg/kg/day) or vehicle only (PBS-Tween-20 (0.1%)/5% DMSO) from three weeks of age for four weeks. Results represent the mean ± S.E from six mice per treatment group and are corrected for β-actin. *p = 0.019; (B) Utrophin protein levels in heart and diaphragm following treatment of *mdx* mice as described in (A) above. Blots were stained with anti-utrophin (MANCHO3; 1∶100) and ECL HRP-conjugated anti-mouse antibody (GE Healthcare). Bands were quantified using Image J and arbitrary units represent utrophin levels corrected for equal loading by α-actinin immunostaining. Results represent a mean ±S.E from eight mice per treatment group except for heart vehicle only which is based on n = 7. *p<0.01; #p<0.05; (C) Immunohistochemical staining of EDL and TA muscle sections (10 µm; 20× magnification) were prepared from *mdx* mice treated as in (A). Sections were stained with anti-utrophin polyclonal antibody URD40 (1∶100) and fluorescein isothiocyanate-conjugated anti-rabbit secondary antibody (1∶1000).

To confirm the *in vivo* activity of SMT C1100, the dystrophin-deficient *mdx* mouse was used to monitor any changes in the dystrophic phenotype after chronic dosing for several weeks. To confirm increases in utrophin expression after repeated daily dosing with SMT C1100, muscle samples were taken for RNA and protein analysis. Fig. 3A demonstrates a two-fold increase in utrophin mRNA as determined by quantitative PCR from *mdx* mice dosed daily with SMT C1100 for 28 days compared to vehicle only. Fig. 3B demonstrates significant increases in utrophin protein quantified from western blots of heart; a muscle notoriously difficult to target with systemic administration of putative DMD therapeutics, and diaphragm; the skeletal muscle most affected in sedentary *mdx* mice. Fig. 3C illustrates a qualitative increase in sarcolemmal-bound utrophin in the tibialis anterior (TA) and EDL muscles after repeat dosing with SMT C1100 following muscle sectioning. A similar result has been observed in both diaphragm and hind limb muscles of forced exercise-treated *mdx* mice (data not shown), suggesting no impact of work load on drug action. This data confirms SMT C1100 drives increased utrophin transcriptional expression *in vivo* and, more importantly, demonstrates increased utrophin staining at the required site of action - the sarcolemma - and independently from muscle work load.

### Benefits of daily dosing of sedentary *mdx* mice with SMT C1100

In the case of the sedentary *mdx* mouse, there is a significant triggering of muscle degeneration at around 4 weeks which continues for a further 4 weeks where limb muscles then appear to reach stasis and levels of regeneration remain stable. One muscle where continued development of necrosis is seen is the diaphragm muscle. The dosing schedule for SMT C1100-treated mice was a single daily administration from day 21 for a further 28 days. This period of dosing encompassed the necrotic degenerative phase resulting from dystrophin deficiency.

**Figure 4:**
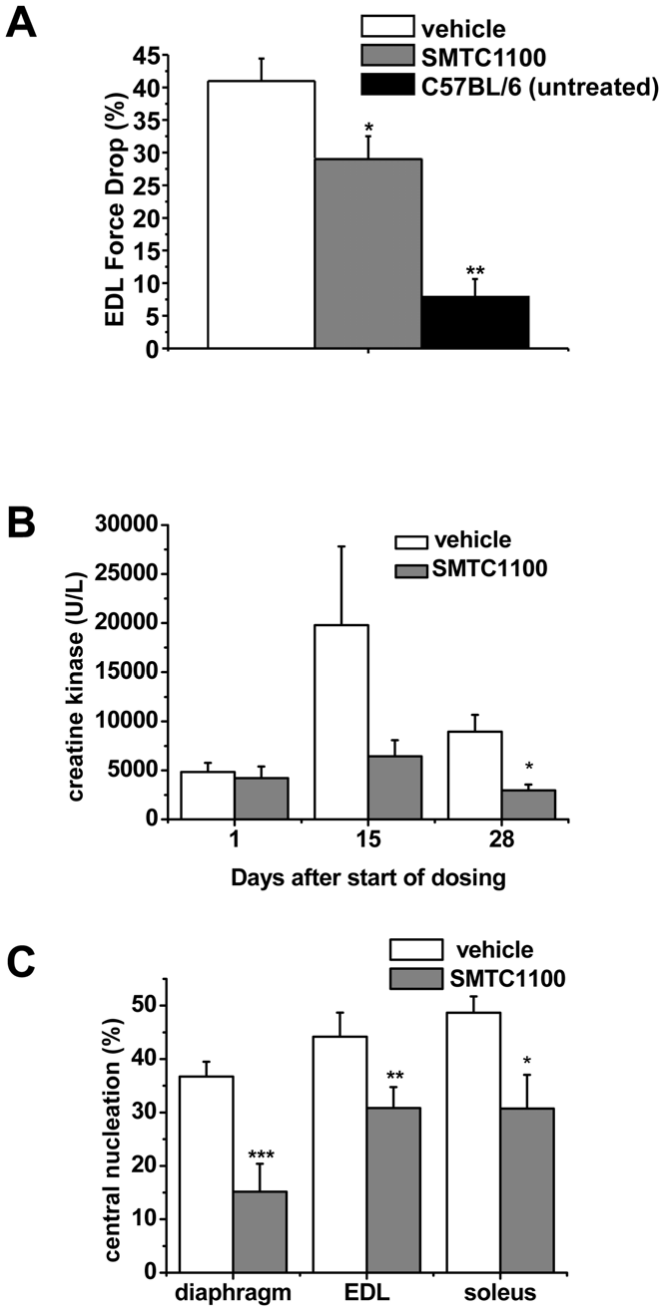
*Ex vivo* analysis of SMT C1100 activity in the *mdx* mouse. (A) *mdx* mice were treated with SMT C1100 (50 mg/kg/day) or vehicle only (0.1% Tween-20/5% DMSO in PBS) via daily i.p. injection from two weeks of age for four weeks. Whilst contracting tetanically, EDL muscles were stretched at 15% of their fibre length. The difference in force produced between the first and fifth stretch is represented as an indicator of the resistance of the muscle to stretch-induced damage. *p<0.05; **p<1.0×10^−5^; (B) levels of serum creatine kinase following oral gavage of *mdx* mice with 50 mg/kg SMT C1100 or vehicle from three weeks of age for four weeks (C) Muscles from the dosing described in (B) were processed to assess the percentage of centrally nucleated fibres *p<0.01; (C)***p = 0.0001; **p = 0.005; *p = 0.003.

The hypothesis to protect myofibres from damage in the absence of dystrophin is that utrophin, if continually localised to the sarcolemma, will replace dystrophin function. If dystrophin negative fibres are protected from damage for longer by the continued presence of utrophin then the catastrophic secondary effects of regeneration, fibrosis and inflammation should be reduced and muscle should be able to function for longer. All of these endpoints are significantly improved in *mdx* dosed daily with SMT C1100 for several weeks compared to *mdx* dosed with vehicle-only. SMT C1100 addresses the primary cause of fibre loss by protecting the sarcolemma from damage as exemplified by increased resistance to eccentric contractions (Fig. 4A) and a reduction in serum creatine kinase levels (Fig. 4B). At the point where the muscle necrosis is at a maximum, SMT C1100 reduces the release of CK into the plasma by 75% compared to vehicle (Fig. 4B; 15 d after the start of dosing). When degeneration has stabilised there is still significant benefit seen as evidenced by continued lower levels of CK (Fig. 4B, 28 d after start of dosing). This data also demonstrates that beneficial effects of SMT C1100 driven utrophin upregulation must occur within a few days after the start of dosing.

The resultant protection of dystrophin-deficient fibres by continued expression of utrophin resulted in a reduction in the level of regeneration taking place in skeletal muscle in *mdx* mice dosed with SMT C1100. This is demonstrated by a significant reduction in the numbers of fibres with centrally localized nuclei, as fibres with peripheral nuclei are thought to be more mature in development and therefore to have been a component of the muscle for longer (Fig. 4C). Significant reduction in centrally nucleated fibres is seen in the skeletal muscles examined including the diaphragm; a more severely affected *mdx* muscle which better mimics the more severe pathology of a DMD patient.

**Figure 5:**
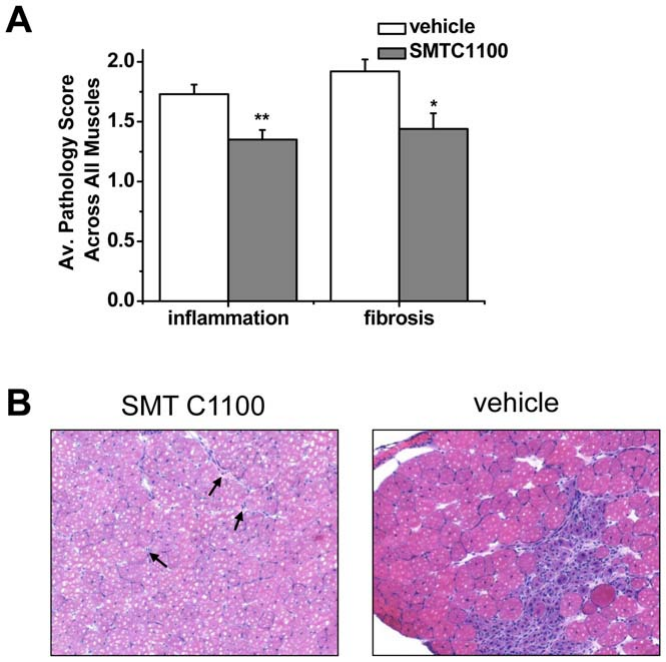
Reduction in secondary pathological features. (A) Data demonstrates the reduction in overall skeletal muscle inflammation and fibrosis from *mdx* treated with SMT C1100 compared to vehicle only. SMT C1100 (50 mg/kg) or vehicle was delivered daily by oral gavage to groups of six *mdx* mice aged around 17 d for a total of 28 days. The TA, EDL, soleus, and diaphragm were removed and five sections from each muscle were stained and analysed blind by a board-certified veterinary pathologist for evidence of inflammation and fibrosis using a standard pathology scoring method described in the methods section. Scoring (0–3) was made for each section from each muscle then averaged for all muscles to give an overall assessment of improvement in the pathological effects of dystrophin deficiency; (B) Qualitative assessment of EDL muscle from SMT C1100-dosed *mdx* scored as 1 = mild - occasional mononuclear inflammatory cells in the inter-bundle connective tissue with focal aggregations of mononuclear inflammatory cells. The arrows mark foci of inflammation. Qualitative assessment of EDL dosed with vehicle and scored as 2 = moderate - multiple foci of mononuclear inflammatory cell infiltration in the inter-bundle connective tissue; occasional mononuclear inflammatory cells between individual muscle fibres.

As the cycle of fibre degeneration and regeneration is being slowed by continued utrophin expression in SMT C1100-dosed *mdx* then the cytoplasmic signals to engage in muscle repair responses such as inflammation and fibrosis are reduced. In normal muscle this inflammatory protection response is dampened down as the proliferation of resident satellite cells fuse and reconnects the broken fibres. However, with the constant degeneration, these protection signals are not switched off, resulting in the continued influx of inflammatory cells and fibroblasts, leading to an increasing cascade of further fibre damage and loss of muscle “space” by fibrotic plaques. Treatment with SMT C1100 significantly reduces this damage by virtue of the reduced fibre regeneration. Blinded analysis by a board-certified veterinary pathologist of muscle sections from *mdx* mice dosed with either vehicle or SMTC1100 demonstrated a significant reduction in both inflammation and fibrosis. Whole muscle sections were rated with a pathology score on a scale from normal (0) – mild (1) – moderate (2) – severe (3). Pooled averages of total scoring from the TA, EDL and soleus are shown (Fig. 5A). A qualitative example of the extent of inflammation from a SMT C1100-dosed EDL or vehicle dosed EDL (Fig. 5B) is shown where the SMT C1100 section was scored as mild (occasional mononuclear inflammatory cells in the inter-bundle connective tissue with focal aggregations of mononuclear inflammatory cells) and the *mdx* as moderate (multiple foci of mononuclear inflammatory cell infiltration in the inter-bundle connective tissue; occasional mononuclear inflammatory cells between individual muscle fibres). This data confirms the concept of reduced fibre damage due to utrophin localization leading to reduced inflammation and fibrosis.

### Benefits of daily dosing of forced exercise *mdx* mice with SMT C1100

A forced exercise regime of chronic exercise was used as a strategy to worsen the murine pathology [14], [21]. Five week old *mdx* mice underwent forced treadmill exercise twice a week and the effects of daily SMT C1100 treatment under this exercise regime were then evaluated.

**Figure 6:**
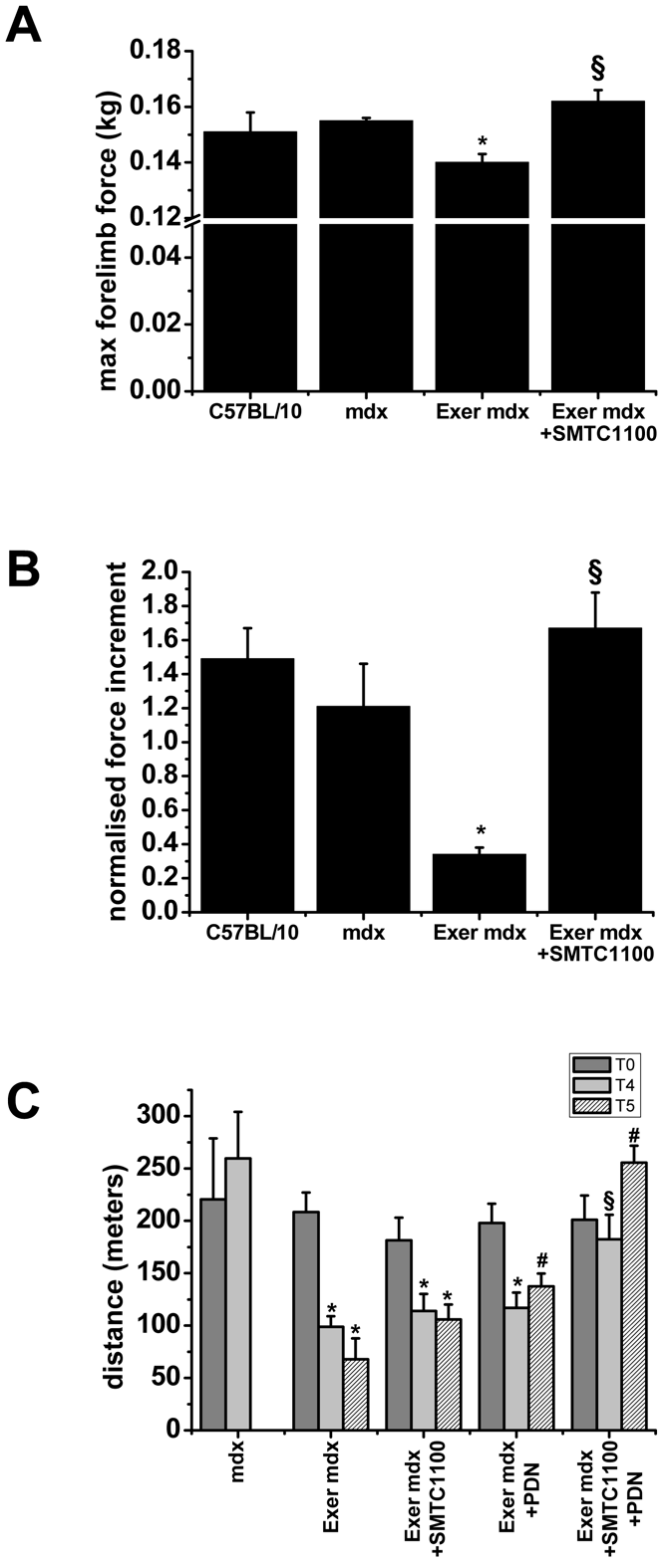
Effect of SMT C1100 on *in vivo* parameters of exercised *mdx* mice. (A) Maximal fore limb strength after 4 weeks of either exercise and/or drug treatment. The values are mean ± SEM from the number of animals shown in each bar (B) Normalized force increment, i.e. difference between the mean values of normalized fore limb strength at time 4 and at time 0. Normalized force values have been calculated for each mouse as the ratio of maximal fore limb strength to mouse body weight. The values are mean ± SE. The SE of ΔNF has been calculated as detailed elsewhere [14], [21]. For (A) and (B) statistical significance between groups was evaluated by ANOVA test for multiple comparison (F-values) and Bonferroni t-test *post hoc* correction. Significantly different versus *sedentary mdx and ^§^Exer *mdx*; p<0.05; (C) Resistance to treadmill running, calculated as the maximal distance the mouse can run when undergoing a single bout of treadmill exercise. The values are mean ± SEM from 3–7 mice and show the maximal distance run (in meters) at T0 (start of forced exercise and dosing) and after four (T4) and five weeks (T5). Statistical significance between groups was evaluated by ANOVA test for multiple comparison (F-values) and Bonferroni t-test *post hoc* correction. Highly significant differences were observed between groups and within groups at the different ages (F>10; p<0.005). The symbols show statistical differences *versus sedentary *mdx* at T4 and #versus vehicle-treated exercised *mdx* at either T4 or T5 (p<0.05 and less).

This exercise protocol significantly worsens *in vivo* parameters readily evaluated by non-invasive approaches, such as fore limb grip and endurance tests. In particular, the exercise protocol induced the typical decrease of fore limb force *in vivo* over time; a reduction which is seldom observed in sedentary *mdx*. SMT C1100-treated *mdx* showed a significant protection against exercise-induced fore limb weakness, as demonstrated by both the maximal strength achieved and the increase in strength after four weeks of dosing (Fig. 6A, B). After four weeks of dosing both values from the SMT C1100-dosed *mdx* were equivalent to those observed in sedentary *mdx* and wild type mice.

Data with direct relevance to DMD treatment was generated using a fatigue assessment of the mice which underwent forced exercise. Fatigue was assessed in an acute endurance test and estimated as the maximal distance run before exhaustion. Sedentary *mdx* mice, although run for a shorter distance than wild type [14], maintain the same exercise performance over time, whilst the exercised *mdx* demonstrate a dramatic increase in fatigability between the start and the fourth and fifth week of training (Fig. 6C). A partial restoration of the resistance to fatigue was observed in SMT C1100-dosed mice, with an increase in distance travelled of around 50% compared to vehicle only after 5 weeks of dosing. Interestingly, this effect was similar to that observed in the exercised *mdx* mice treated with PDN; which is currently the gold standard in clinical treatment for Duchenne patients. Significant synergy was observed when SMT C1100 was co-administered with PDN for five weeks. The forced exercise *mdx* were completely resistant to fatigue and were able to continue running as far as the sedentary *mdx* (Fig. 6C). This equated to an increase in distance travelled of around 350% compared to the vehicle-treated forced exercise *mdx*.

**Figure 7:**
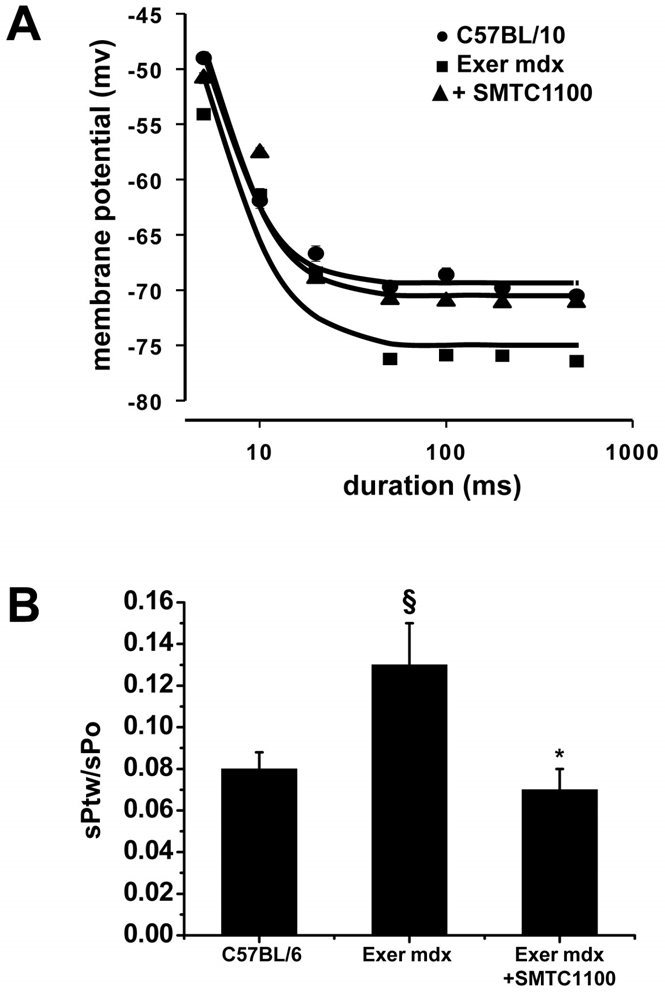
Effect of SMT C1100 treatment on calcium-dependent functional parameters of exercised *mdx* muscles. (A) Strength-duration curves describing the mechanical threshold of treated and/or untreated EDL muscle fibers. The values are means ±S.E.M. (from 10–45 fibers from 3–5 muscles) of the membrane potential at which contraction occurs in response to a depolarizing voltage step of variable duration (5–500 ms) by means of two microelectrode “point” voltage clamp method. The goodness-of-fit has been estimated by χ^2^ analysis. Error bars are sometime not detectable if smaller than symbol size. Although not shown for graphical reasons, the threshold values of C1100 treated myofibres are significantly different with respect to those of untreated ones, at each pulse duration (0.001<p<0.05 by Student's t test); (B) ratio between twitch (sPtw) and tetanic tension, as mean ±S.E.M. from 5–7 EDL muscles. Significantly different *versus Exer *mdx*; p<0.05 and §versus sedentary *mdx*; p<0.025.

*Ex vivo* analysis on isolated muscles from forced exercise *mdx* mice demonstrated that SMT C1100 exerted a significant amelioration of calcium-dependent functional parameters. These are typically modified in *mdx* muscles due to the altered calcium homeostasis, which in turn is believed to drive the rate of degeneration. In SMT C1100-treated EDL muscle fibres the strength-duration curve describing the mechanical threshold was significantly shifted toward the more positive membrane potential values and almost overlapped with that observed in wildtype myofibres (Fig. 7A). The rheobase value of SMT C1100-treated muscles approached the wild type value (−69.3±0.4 mV), and was approximately 5 mV less negative than that of non-treated exercised group (−70.5±1.2 mV vs. −75±1.5 mV: p<0.05 by Student's t test). Interestingly, this parameter is not ameliorated by a partial increase in dystrophin expression by gentamicin treatment [21]. Similarly, the ratio between twitch and tetanic tension was significantly reduced in SMT C1100-treated exercised *mdx* EDL muscles with respect to untreated counterparts, again demonstrating that SMT C1100 treatment generates similar values to those typically found in wild type EDL muscle (Fig. 7B). The amelioration of calcium-dependent parameters was paralleled by a partial, although significant, 18% decrease in the cytosolic Ca^2+^ level, as determined by fura-2 microspectrofluorimetry [14] (data not shown), thus corroborating that the sarcolemmal bound utrophin stimulated by SMT C1100 treatment can improve calcium-mediate mechanotransduction signalling.

## Discussion

This manuscript illustrates the effectiveness of dosing a well-established mouse model of DMD with a novel oral utrophin upregulator for several weeks. SMT C1100 induces increased levels of utrophin RNA in human muscle cells and significantly reduces dystrophin-deficient muscle pathology to such an extent that significant benefit on whole body strength and endurance is observed. Currently PDN and deflazacort are the only drugs approved by the regulatory authorities for the treatment of DMD. We believe that fatigue testing of *mdx* after a regime of forced exercise is a good surrogate for the primary clinical endpoint which will be used in DMD trials, *i.e.* in the 6MWD. Dosing with SMT C1100 alone demonstrated significant benefit in this surrogate model, and the 50% increase in the distance walked would have achieved the required efficacy endpoint if translated over to the 6MWD in DMD trials. Combining doses of SMT C1100 and PDN for several weeks completely prevented fatigue in this model. Thus, the combination of the two drugs with presumed different modes of action protect the muscle from fatiguing with exercise, thereby allowing for significantly increased ambulation. High levels of long term steroid use have unwanted side effects, however a steroid sparing therapy (either reducing dose or frequency to alleviate the unwanted side effects) working synergistically with a utrophin upregulator, has the potential to become the new standard of care for all DMD patients.

These results show proof-of-principle for the development of small molecules able to increase levels of utrophin for the therapy of DMD. The great advantage of this approach is that it will be possible to treat all DMD and Becker patients, irrespective of their dystrophin mutation. In addition, it could also be used in combination with existing/novel strategies in the future, including utrophin stabilisation strategies such as biglycan.

In choosing a dosing route, an orally bioavailable product to be taken at home would be the ideal preference. In short, SMT C1100 has the perfect profile - an oral drug suitable for treating all DMD patients. In the recent clinical trial sponsored by BioMarin, after repeat dosing SMT C1100 (BMN-195) achieved low plasma exposure. This is frequently a problem in Phase I trials and issues of low exposure can often be addressed by developing new formulations of the drug to increase bioavailability. From the data presented here, only modest plasma levels of around 0.5 µM SMT C1100 maintained over several hours are sufficient to generate enough utrophin for substantial benefit. This strongly supports the importance of retesting new formulations of SMT C1100 in new Phase I clinical trials with a view to progressing to DMD patient trials.
